# Supplementary material for: Associations of age at diagnosis of breast cancer with incident myocardial infarction and heart failure: A prospective cohort study
Source: eLife. 2024 Aug 22;13:RP95901. doi: 10.7554/eLife.95901 (PMC11341089; doi:10.7554/eLife.95901)
Supplement: Supplementary file 1. — (A) STROBE Statement—Checklist of items that should be included in reports of cohort studies. (B) Ascertainment of breast cancer and age at breast cancer diagnosis. (C) Ascertainment of myocardial infarction and heart failure. (D) Definition and assessment of covariates. (E) Baseline characteristics of participants by breast cancer status after propensity score matching (n = 64,964). (F) Associations of age at breast cancer diagnosis with incident myocardial infarction and heart failure among breast cancer participants: competing risk models (n = 16,241). (G) Associations of breast cancer with incident myocardial infarction and heart failure among different diagnosis age groups after propensity score matching: competing risk models (n = 64,964). (H) Associations of age at breast cancer diagnosis with incident myocardial infarction and heart failure among breast cancer participants after excluding myocardial infarction and heart failure diagnosed within 5 years since baseline (n = 15,589). (I) Associations of breast cancer with incident myocardial infarction and heart failure among different diagnosis age groups after excluding myocardial infarction and heart failure diagnosed within 5 years since baseline, results from propensity score matching analyses (n = 62,356). (J) Associations of age at breast cancer diagnosis with incident myocardial infarction and heart failure among breast cancer participants after excluding participants aged <50 years at baseline (n = 14,000). (K) Associations of breast cancer with incident myocardial infarction and heart failure among different diagnosis age groups after excluding participants aged <50 years at baseline, results from propensity score matching analyses (n = 56,000). (L) Associations of age at breast cancer diagnosis with incident myocardial infarction and heart failure among breast cancer participants when the follow-up period ends on December 31, 2019 (n = 15,909). (M) Associations of breast cancer with incident myocardi [file elife-95901-supp1.docx]

**Supplementary File 1**

**Supplementary file 1A. STROBE Statement—Checklist of items that should be included in reports of cohort studies.**

|  | Item No | Recommendation | Yes/No |
| --- | --- | --- | --- |
| **Title and abstract** | 1 | (*a*) Indicate the study’s design with a commonly used term in the title or the abstract | Yes |
|  |  | (*b*) Provide in the abstract an informative and balanced summary of what was done and what was found | Yes |
| **Introduction** | | |  |
| Background/rationale | 2 | Explain the scientific background and rationale for the investigation being reported | Yes |
| Objectives | 3 | State specific objectives, including any prespecified hypotheses | Yes |
| **Methods** | | |  |
| Study design | 4 | Present key elements of study design early in the paper | Yes |
| Setting | 5 | Describe the setting, locations, and relevant dates, including periods of recruitment, exposure, follow-up, and data collection | Yes |
| Participants | 6 | (*a*) Give the eligibility criteria, and the sources and methods of selection of participants. Describe methods of follow-up | Yes |
|  |  | (*b*) For matched studies, give matching criteria and number of exposed and unexposed | Yes |
| Variables | 7 | Clearly define all outcomes, exposures, predictors, potential confounders, and effect modifiers. Give diagnostic criteria, if applicable | Yes |
| Data sources/ measurement | 8* | For each variable of interest, give sources of data and details of methods of assessment (measurement). Describe comparability of assessment methods if there is more than one group | Yes |
| Bias | 9 | Describe any efforts to address potential sources of bias | Yes |
| Study size | 10 | Explain how the study size was arrived at | No |
| Quantitative variables | 11 | Explain how quantitative variables were handled in the analyses. If applicable, describe which groupings were chosen and why | Yes |
| Statistical methods | 12 | (*a*) Describe all statistical methods, including those used to control for confounding | Yes |
|  |  | (*b*) Describe any methods used to examine subgroups and interactions | Yes |
|  |  | (*c*) Explain how missing data were addressed | Yes |
|  |  | (*d*) If applicable, explain how loss to follow-up was addressed | No |
|  |  | (*e*) Describe any sensitivity analyses | Yes |
| **Results** | | |  |
| Participants | 13* | (a) Report numbers of individuals at each stage of study—eg numbers potentially eligible, examined for eligibility, confirmed eligible, included in the study, completing follow-up, and analysed | Yes |
|  |  | (b) Give reasons for non-participation at each stage | Yes |
|  |  | (c) Consider use of a flow diagram | Yes |
| Descriptive data | 14* | (a) Give characteristics of study participants (eg demographic, clinical, social) and information on exposures and potential confounders | Yes |
|  |  | (b) Indicate number of participants with missing data for each variable of interest | Yes |
|  |  | (c) Summarise follow-up time (eg, average and total amount) | Yes |
| Outcome data | 15* | Report numbers of outcome events or summary measures over time | Yes |
| Main results | 16 | (*a*) Give unadjusted estimates and, if applicable, confounder-adjusted estimates and their precision (eg, 95% confidence interval). Make clear which confounders were adjusted for and why they were included | Yes |
|  |  | (*b*) Report category boundaries when continuous variables were categorized | Yes |
|  |  | (*c*) If relevant, consider translating estimates of relative risk into absolute risk for a meaningful time period | No |
| Other analyses | 17 | Report other analyses done—eg analyses of subgroups and interactions, and sensitivity analyses | Yes |
| **Discussion** | | |  |
| Key results | 18 | Summarise key results with reference to study objectives | Yes |
| Limitations | 19 | Discuss limitations of the study, taking into account sources of potential bias or imprecision. Discuss both direction and magnitude of any potential bias | Yes |
| Interpretation | 20 | Give a cautious overall interpretation of results considering objectives, limitations, multiplicity of analyses, results from similar studies, and other relevant evidence | Yes |
| Generalisability | 21 | Discuss the generalisability (external validity) of the study results | Yes |
| **Other information** | | |  |
| Funding | 22 | Give the source of funding and the role of the funders for the present study and, if applicable, for the original study on which the present article is based | Yes |

*Give information separately for exposed and unexposed groups.

**Note:** An Explanation and Elaboration article discusses each checklist item and gives methodological background and published examples of transparent reporting. The STROBE checklist is best used in conjunction with this article (freely available on the Web sites of PLoS Medicine at http://www.plosmedicine.org/, Annals of Internal Medicine at http://www.annals.org/, and Epidemiology at http://www.epidem.com/). Information on the STROBE Initiative is available at http://www.strobe-statement.org.

**Supplementary file 1B. Ascertainment of breast cancer and age at breast cancer diagnosis.**

|  | **Definition** | **Assessment** | **UK biobank Data-Field ID** |
| --- | --- | --- | --- |
| Breast cancer | Yes, No | (1) Cancer register: breast cancer (the International Classification of Diseases Tenth Revision [ICD**–**10] codes of C50). | 40006 |
| Breast cancer diagnosis age | Years | (1) Cancer register: age at cancer diagnosis (the International Classification of Diseases Tenth Revision [ICD**–**10] codes of C50). | 40008 |

**Supplementary file 1C. Ascertainment of myocardial infarction and heart failure.**

| **Outcomes** | **Definition** | **Assessment** | **UK biobank Data-Field ID** |
| --- | --- | --- | --- |
| Myocardial infarction | Yes, No | (1) Algorithmically-defined outcomes: date of myocardial infarction, STEMI, and NSTEMI. | 42000, 42002, 42004 |
| Heart failure | Yes, No | (1) First occurrences of circulatory system disorders: heart failure (the International Classification of Diseases Tenth Revision [ICD**–**10] codes of I50). | 131354 |

**Supplementary file 1D. Definition and assessment of covariates.**

| **Covariates** | **Definition** | **Assessment** | **UK biobank Data-Field ID** |
| --- | --- | --- | --- |
| Age (years) | Age in years | Difference between date attended baseline assessment and date of birth recorded by NHS | 21003 |
| Ethnicity | White, Nonwhite (Mixed, Asian, Black, Chinese, Other) | Touchscreen questionnaire: “What is your ethnic group?” | 21000 |
| Education | Higher education (college or university degree, other professional qualifications), other than higher education | Touchscreen questionnaire: “Which of the following qualifications do you have?” | 6138 |
| Current smoking | Yes, No | Touchscreen questionnaire: “Do you smoke tobacco now?” and “In the past, how often have you smoked tobacco?” | 20116 |
| Current drinking | At least once per week, less than once per week | Touchscreen questionnaire: “About how often do you drink alcohol?” | 1558 |
| Obesity | Yes, No | Physical measures: body mass index (BMI) ≥30 kg/m^2^ | 23104 |
| Exercise | Attending moderate or vigorous physical activity 10+ minutes at least twice per week, less than twice per week | Touchscreen questionnaire: “In a typical WEEK, on how many days did you do 10 minutes or more of moderate physical activities like carrying light loads, cycling at normal pace? (Do not include walking);  In a typical WEEK, how many days did you do 10 minutes or more of vigorous physical activity? (These are activities that make you sweat or breathe hard such as fast cycling, aerobics, heavy lifting)” | 884, 904 |
| Low-density lipoprotein cholesterol | mmol/L | Blood biochemistry: Low-density lipoprotein cholesterol | 30780 |
| Depressed mood | Yes (nearly every day or more than half the days), No (not at all or several days) | Touchscreen questionnaire: “Over the past two weeks, how often have you felt down, depressed or hopeless?” | 2050 |
| Hypertension | Yes, No | Touchscreen questionnaire and verbal interview: self-reported hypertension or antihypertensive drug use;  Average SBP/DBP ≥ 140/90 mmHg at baseline | 6150, 20002, 6177, 4079, 4080, 93, 94 |
| Diabetes | Yes, No | Touchscreen questionnaire and verbal interview: self-reported diabetes (diabetes, type 1 diabetes, or type 2 diabetes) or antidiabetic drug use;  Plasma HbA_1c_ ≥ 48 mmol/mol (6.5%) | 2443, 20002, 6153, 6177, 30750, 20003 |
| Statin use | Yes, No | Verbal interview: self-reported statin use | 20003 |
| Antihypertensive drug use | Yes, No | Touchscreen questionnaire and verbal interview: self-reported antihypertensive drug use | 6153, 6177, 20003 |
| Antidiabetic drug use | Yes, No | Touchscreen questionnaire and verbal interview: self-reported antidiabetic drug use | 6153, 6177, 20003 |

**Supplementary file 1E. Baseline characteristics of participants by breast cancer status after propensity score matching (n=64 964).**

| **Characteristic** | **Breast cancer** | **Non-breast cancer** | **Effect size**^a^ |
| --- | --- | --- | --- |
| **<50 years (n=10 696, median follow-up=13 years, interquartile range: 12–13 years)** | | | |
| Age, years | 52.1±7.4 | 52.1±7.4 | –0.001 |
| White | 2 514 (94.0) | 7 563 (94.3) | –0.005 |
| Higher education | 1 382 (51.7) | 4 139 (51.6) | 0.001 |
| Current smoking | 257 (9.6) | 756 (9.4) | 0.003 |
| Current drinking | 1 727 (64.6) | 5 303 (66.1) | –0.014 |
| Obesity | 505 (18.9) | 1 507 (18.8) | 0.001 |
| Exercise | 2 048 (76.6) | 6 132 (76.4) | 0.002 |
| LDL-C, mmol/L | 3.53±0.88 | 3.52±0.84 | 0.022 |
| Depressed mood | 174 (6.5) | 498 (6.2) | 0.005 |
| Hypertension | 976 (36.5) | 2 938 (36.6) | –0.001 |
| Diabetes | 90 (3.4) | 250 (3.1) | 0.006 |
| Antihypertensive drug use | 272 (10.2) | 795 (9.9) | 0.004 |
| Antidiabetic drug use | 54 (2.0) | 154 (1.9) | 0.003 |
| Statin use | 168 (6.3) | 485 (6.1) | 0.004 |
| **50–59 years (n=22 548, median follow-up=13 years, interquartile range: 12–13 years)** | | | |
| Age, years | 56.7±7.2 | 56.7±7.8 | 0.003 |
| White | 5 416 (96.1) | 16 252 (96.1) | –0.001 |
| Higher education | 2 767 (49.1) | 8 417 (49.8) | –0.006 |
| Current smoking | 530 (9.4) | 1 588 (9.4) | <0.001 |
| Current drinking | 3 650 (64.8) | 10 925 (64.6) | 0.001 |
| Obesity | 1 339 (23.8) | 3 949 (23.4) | 0.004 |
| Exercise | 4 334 (76.9) | 13 056 (77.2) | –0.003 |
| LDL-C, mmol/L | 3.64±0.88 | 3.66±0.87 | –0.020 |
| Depressed mood | 287 (5.1) | 867 (5.1) | –0.001 |
| Hypertension | 2 686 (47.7) | 7 908 (46.8) | 0.008 |
| Diabetes | 261 (4.6) | 754 (4.5) | 0.004 |
| Antihypertensive drug use | 900 (16.0) | 2 662 (15.7) | 0.003 |
| Antidiabetic drug use | 156 (2.8) | 459 (2.7) | 0.001 |
| Statin use | 536 (9.5) | 1 620 (9.6) | –0.001 |
| **≥60 years (n=31 720, median follow-up=13 years, interquartile range: 12–14 years)** | | | |
| Age, years | 62.7±4.7 | 62.7±4.8 | –0.004 |
| White | 7 689 (97.0) | 23 057 (96.9) | 0.001 |
| Higher education | 3 422 (43.2) | 10 349 (43.5) | –0.003 |
| Current smoking | 603 (7.6) | 1 780 (7.5) | 0.002 |
| Current drinking | 5 074 (64.0) | 15 275 (64.2) | –0.002 |
| Obesity | 2 090 (26.4) | 6 184 (26.0) | 0.004 |
| Exercise | 6 124 (77.2) | 18 493 (77.7) | –0.005 |
| LDL-C, mmol/L | 3.74±0.88 | 3.74±0.88 | –0.006 |
| Depressed mood | 317 (4.0) | 932 (3.9) | 0.002 |
| Hypertension | 4 981 (62.8) | 14 791 (62.2) | 0.006 |
| Diabetes | 444 (5.6) | 1 299 (5.5) | 0.003 |
| Antihypertensive drug use | 2 031 (25.6) | 6 024 (25.3) | 0.003 |
| Antidiabetic drug use | 239 (3.0) | 722 (3.0) | –0.001 |
| Statin use | 1 181 (14.9) | 3 665 (15.4) | –0.006 |

The results are presented as the mean ± standard deviation, or No. (%).

^a^The effect sizes are standardized mean differences for continuous outcomes and the Phi coefficient for dichotomous outcomes.

LDL-C, low-density lipoprotein cholesterol.

**Supplementary file 1F. Associations of age at breast cancer diagnosis with incident myocardial infarction and heart failure among participants with breast cancer: competing risk models (n=16 241).**

| **Outcome** | **HR (95% CI)**^a^ | ***P* value** |
| --- | --- | --- |
| Myocardial infarction |  |  |
| ≥60 years (n=7 930) | Reference | / |
| 50–59 years (n=5 637) | 1.02 (0.76 to 1.37) | 0.896 |
| <50 years (n=2 674) | 2.11 (1.47 to 3.04) | <0.001 |
| Per 10-year decrease | 1.33 (1.16 to 1.53) | <0.001 |
| Heart failure |  |  |
| ≥60 years (n=7 930) | Reference | / |
| 50–59 years (n=5 637) | 1.29 (1.04 to 1.59) | 0.019 |
| <50 years (n=2 674) | 1.62 (1.18 to 2.23) | 0.003 |
| Per 10-year decrease | 1.28 (1.15 to 1.43) | <0.001 |

^a^Adjusted for age, ethnicity, education, current smoking, current drinking, obesity, exercise, low-density lipoprotein cholesterol, depressed mood, hypertension, diabetes, antihypertensive drug use, antidiabetic drug use, and statin use.

HR, hazard ratio; CI, confidence interval.

**Supplementary file 1G. Associations of breast cancer with incident myocardial infarction and heart failure among different diagnosis age groups after propensity score matching: competing risk models (n=64 964).**

| **Outcome** | **HR (95% CI)**^a^  **Breast cancer vs. Non-breast cancer** | ***P* value** |
| --- | --- | --- |
| Myocardial infarction |  |  |
| ≥60 years (n=31 720) | 0.73 (0.61 to 0.87) | <0.001 |
| 50–59 years (n=22 548) | 0.71 (0.55 to 0.92) | 0.010 |
| <50 years (n=10 696) | 1.64 (1.14 to 2.37) | 0.008 |
| Heart failure |  |  |
| ≥60 years (n=31 720) | 1.01 (0.88 to 1.17) | 0.885 |
| 50–59 years (n=22 548) | 1.31 (1.07 to 1.60) | 0.008 |
| <50 years (n=10 696) | 2.09 (1.45 to 2.99) | <0.001 |

^a^Adjusted for age, ethnicity, education, current smoking, current drinking, obesity, exercise, low-density lipoprotein cholesterol, depressed mood, hypertension, diabetes, antihypertensive drug use, antidiabetic drug use, and statin use.

HR, hazard ratio; CI, confidence interval.

**Supplementary file 1H. Associations of age at breast cancer diagnosis with incident myocardial infarction and heart failure among participants with breast cancer after excluding myocardial infarction and heart failure diagnosed within 5 years since baseline (n=15 589).**

| **Outcome** | **HR (95% CI)**^a^ | ***P* value** |
| --- | --- | --- |
| Myocardial infarction |  |  |
| ≥60 years (n=7 722) | Reference | / |
| 50–59 years (n=5 368) | 0.80 (0.58 to 1.12) | 0.196 |
| <50 years (n=2 499) | 1.58 (1.03 to 2.43) | 0.038 |
| Per 10-year decrease | 1.13 (0.97 to 1.32) | 0.131 |
| Heart failure |  |  |
| ≥60 years (n=7 722) | Reference | / |
| 50–59 years (n=5 368) | 1.24 (0.99 to 1.56) | 0.066 |
| <50 years (n=2 499) | 1.45 (1.00 to 2.09) | 0.049 |
| Per 10-year decrease | 1.21 (1.07 to 1.37) | 0.002 |

^a^Adjusted for age, ethnicity, education, current smoking, current drinking, obesity, exercise, low-density lipoprotein cholesterol, depressed mood, hypertension, diabetes, antihypertensive drug use, antidiabetic drug use, and statin use.

HR, hazard ratio; CI, confidence interval.

**Supplementary file 1I. Associations of breast cancer with incident myocardial infarction and heart failure among different diagnosis age groups after excluding myocardial infarction and heart failure diagnosed within 5 years since baseline, results from propensity score matching analyses (n=62 356).**

| **Outcome** | **HR (95% CI)**^a^  **Breast cancer vs. Non-breast cancer** | ***P* value** |
| --- | --- | --- |
| Myocardial infarction |  |  |
| ≥60 years (n=30 888) | 0.86 (0.71 to 1.03) | 0.108 |
| 50–59 years (n=21 472) | 0.65 (0.48 to 0.88) | 0.005 |
| <50 years (n=9 996) | 1.16 (0.76 to 1.77) | 0.494 |
| Heart failure |  |  |
| ≥60 years (n=30 888) | 1.19 (1.02 to 1.40) | 0.028 |
| 50–59 years (n=21 472) | 1.37 (1.10 to 1.71) | 0.005 |
| <50 years (n=9 996) | 2.49 (1.61 to 3.85) | <0.001 |

^a^Adjusted for age, ethnicity, education, current smoking, current drinking, obesity, exercise, low-density lipoprotein cholesterol, depressed mood, hypertension, diabetes, antihypertensive drug use, antidiabetic drug use, and statin use.

HR, hazard ratio; CI, confidence interval.

**Supplementary file 1J. Associations of age at breast cancer diagnosis with incident myocardial infarction and heart failure among participants with breast cancer after excluding participants aged <50 years at baseline (n=14 000).**

| **Outcome** | **HR (95% CI)**^a^ | ***P* value** |
| --- | --- | --- |
| Myocardial infarction |  |  |
| ≥60 years (n=7 907) | Reference | / |
| 50–59 years (n=4 533) | 1.08 (0.81 to 1.44) | 0.611 |
| <50 years (n=1 560) | 2.08 (1.42 to 3.04) | <0.001 |
| Per 10-year decrease | 1.35 (1.17 to 1.55) | <0.001 |
| Heart failure |  |  |
| ≥60 years (n=7 907) | Reference | / |
| 50–59 years (n=4 533) | 1.32 (1.06 to 1.64) | 0.012 |
| <50 years (n=1 560) | 1.68 (1.20 to 2.36) | 0.003 |
| Per 10-year decrease | 1.31 (1.18 to 1.46) | <0.001 |

^a^Adjusted for age, ethnicity, education, current smoking, current drinking, obesity, exercise, low-density lipoprotein cholesterol, depressed mood, hypertension, diabetes, antihypertensive drug use, antidiabetic drug use, and statin use.

HR, hazard ratio; CI, confidence interval.

**Supplementary file 1K. Associations of breast cancer with incident myocardial infarction and heart failure among different diagnosis age groups after excluding participants aged <50 years at baseline, results from propensity score matching analyses (n=56 000).**

| **Outcome** | **HR (95% CI)**^a^  **Breast cancer vs. Non-breast cancer** | ***P* value** |
| --- | --- | --- |
| Myocardial infarction |  |  |
| ≥60 years (n=31 628) | 0.80 (0.68 to 0.96) | 0.013 |
| 50–59 years (n=18 132) | 0.84 (0.65 to 1.10) | 0.201 |
| <50 years (n=6 240) | 1.60 (1.07 to 2.39) | 0.022 |
| Heart failure |  |  |
| ≥60 years (n=31 628) | 1.04 (0.90 to 1.20) | 0.627 |
| 50–59 years (n=18 132) | 1.30 (1.06 to 1.60) | 0.013 |
| <50 years (n=6 240) | 1.58 (1.08 to 2.30) | 0.018 |

^a^Adjusted for age, ethnicity, education, current smoking, current drinking, obesity, exercise, low-density lipoprotein cholesterol, depressed mood, hypertension, diabetes, antihypertensive drug use, antidiabetic drug use, and statin use.

HR, hazard ratio; CI, confidence interval.

**Supplementary file 1L. Associations of age at breast cancer diagnosis with incident myocardial infarction and heart failure among participants with breast cancer when the follow-up period ends on December 31, 2019 (n=15 909).**

| **Outcome** | **HR (95% CI)**^a^ | ***P* value** |
| --- | --- | --- |
| Myocardial infarction |  |  |
| ≥60 years (n=7 672) | Reference | / |
| 50–59 years (n=5 563) | 1.31 (0.94 to 1.82) | 0.109 |
| <50 years (n=2 674) | 2.52 (1.66 to 3.84) | <0.001 |
| Per 10-year decrease | 1.51 (1.29 to 1.76) | <0.001 |
| Heart failure |  |  |
| ≥60 years (n=7 672) | Reference | / |
| 50–59 years (n=5 563) | 1.56 (1.22 to 1.99) | <0.001 |
| <50 years (n=2 674) | 1.77 (1.22 to 2.58) | 0.003 |
| Per 10-year decrease | 1.38 (1.22 to 1.57) | <0.001 |

^a^Adjusted for age, ethnicity, education, current smoking, current drinking, obesity, exercise, low-density lipoprotein cholesterol, depressed mood, hypertension, diabetes, antihypertensive drug use, antidiabetic drug use, and statin use.

HR, hazard ratio; CI, confidence interval.

**Supplementary file 1M. Associations of breast cancer with incident myocardial infarction and heart failure among different diagnosis age groups when the follow-up period ends on December 31, 2019, results from propensity score matching analyses (n=63 636).**

| **Outcome** | **HR (95% CI)**^a^  **Breast cancer vs. Non-breast cancer** | ***P* value** |
| --- | --- | --- |
| Myocardial infarction |  |  |
| ≥60 years (n=30 688) | 0.62 (0.50 to 0.76) | <0.001 |
| 50–59 years (n=22 252) | 0.82 (0.61 to 1.08) | 0.158 |
| <50 years (n=10 696) | 1.21 (0.81 to 1.79) | 0.352 |
| Heart failure |  |  |
| ≥60 years (n=30 688) | 0.86 (0.73 to 1.02) | 0.088 |
| 50–59 years (n=22 252) | 1.57 (1.25 to 1.96) | <0.001 |
| <50 years (n=10 696) | 1.37 (0.93 to 2.02) | 0.115 |

^a^Adjusted for age, ethnicity, education, current smoking, current drinking, obesity, exercise, low-density lipoprotein cholesterol, depressed mood, hypertension, diabetes, antihypertensive drug use, antidiabetic drug use, and statin use.

HR, hazard ratio; CI, confidence interval.

**Supplementary file 1N. Associations of age at breast cancer diagnosis with incident myocardial infarction and heart failure among participants with breast cancer after further adjusting for menopausal status, breast cancer surgery, and hormone replacement therapy (n=16 241).**

| **Outcome** | **HR (95% CI)**^a^ | ***P* value** | **Number (%)** | | |
| --- | --- | --- | --- | --- | --- |
|  |  |  | **Menopause** | **Surgery** | **HRT** |
| Myocardial infarction |  |  |  |  |  |
| ≥60 years (n=7 672) | Reference | / | 6 503 (82.0%) | 7 453 (94.0%) | 4 362 (55.0%) |
| 50–59 years (n=5 563) | 1.05 (0.78 to 1.41) | 0.744 | 3 535 (62.7%) | 4 789 (85.0%) | 2 134 (37.9%) |
| <50 years (n=2 674) | 2.19 (1.53 to 3.16) | <0.001 | 1 422 (53.2%) | 1 983 (74.2%) | 288 (10.8 %) |
| Per 10-year decrease | 1.36 (1.19 to 1.56) | <0.001 | 11 460 (70.6 %) | 14 225 (87.6%) | 6 784 (41.8%) |
| Heart failure |  |  |  |  |  |
| ≥60 years (n=7672) | Reference | / | 6 503 (82.0%) | 7 453 (94.0%) | 4 362 (55.0%) |
| 50–59 years (n=5563) | 1.33 (1.08 to 1.64) | 0.008 | 3 535 (62.7%) | 4 789 (85.0%) | 2 134 (37.9%) |
| <50 years (n=2674) | 1.74 (1.25 to 2.41) | 0.001 | 1 422 (53.2%) | 1 983 (74.2%) | 288 (10.8 %) |
| Per 10-year decrease | 1.33 (1.19 to 1.48) | <0.001 | 11 460 (70.6 %) | 14 225 (87.6%) | 6 784 (41.8%) |

^a^Adjusted for age, ethnicity, education, current smoking, current drinking, obesity, exercise, low-density lipoprotein cholesterol, depressed mood, hypertension, diabetes, antihypertensive drug use, antidiabetic drug use, statin use, menopausal status, breast cancer surgery, and hormone replacement therapy.

HR, hazard ratio; CI, confidence interval.

**Supplementary file 1O. Associations of breast cancer with incident myocardial infarction and heart failure among different diagnosis age groups after further adjusting for menopausal status, breast cancer surgery, and hormone replacement therapy, results from propensity score matching analyses (n=64 964).**

| **Outcome** | **HR (95% CI)**^a^  **Breast cancer vs. Non-breast cancer** | ***P* value** |
| --- | --- | --- |
| Myocardial infarction |  |  |
| ≥60 years (n=30 688) | 0.75 (0.63 to 0.89) | 0.001 |
| 50–59 years (n=22 252) | 0.75 (0.58 to 0.98) | 0.032 |
| <50 years (n=10 696) | 1.47 (1.03 to 2.61) | 0.036 |
| Heart failure |  |  |
| ≥60 years (n=30 688) | 1.03 (0.89 to 1.19) | 0.669 |
| 50–59 years (n=22 252) | 1.39 (1.13 to 1.69) | 0.001 |
| <50 years (n=10 696) | 2.18 (1.48 to 3.20) | <0.001 |

^a^Adjusted for age, ethnicity, education, current smoking, current drinking, obesity, exercise, low-density lipoprotein cholesterol, depressed mood, hypertension, diabetes, antihypertensive drug use, antidiabetic drug use, statin use, menopausal status, breast cancer surgery, and hormone replacement therapy.

HR, hazard ratio; CI, confidence interval.

**Supplementary file 1P. Comparison of baseline characteristics between participants included (n=251 277) and excluded due to history of myocardial infarction or heart failure, without complete data on low-density lipoprotein cholesterol, or having myocardial infarction or heart failure before breast cancer at follow-ups (n=22 048).**

| **Characteristics** | **Participants included**  **(n=251 277)** | **Participants excluded**  **(n=22 048)** | **Effect size**^a^ |
| --- | --- | --- | --- |
| Age, years | 56.8±8.0 | 57.5±8.0 | –0.085 |
| White | 420 508 (94.1) | 51 535 (92.5) | 0.029 |
| Higher education | 116 933 (46.5) | 8 551 (38.8) | 0.042 |
| Current smoking | 22 154 (8.8) | 2 207 (10.0) | –0.011 |
| Current drinking | 157 130 (62.5) | 12 775 (57.9) | 0.026 |
| Obesity | 58 255 (23.2) | 6 042 (27.4) | –0.027 |
| Exercise | 195 358 (77.8) | 16 473 (74.7) | 0.020 |
| Depressed mood | 13 129 (5.2) | 1 382 (6.3) | –0.013 |
| Hypertension | 121 310 (48.3) | 11 324 (51.4) | –0.017 |
| Diabetes | 10 678 (4.3) | 1 530 (6.9) | –0.036 |
| LDL-C, mmol/L | 3.63±0.87 | 2.99±0.86 | 0.739 |
| Antihypertensive drug use | 42 970 (17.1) | 4 943 (22.4) | –0.038 |
| Antidiabetic drug use | 6 077 (2.4) | 924 (4.2) | –0.031 |
| Statin use | 26 387 (10.5) | 4 033 (18.3) | –0.068 |

The results are presented as the mean ± standard deviation, or No. (%).

^a^Calculated by using a *t* test, or Chi-square test.

LDL-C, low-density lipoprotein cholesterol.
